# Changes in Poultry Handling Behavior and Poultry Mortality Reporting among Rural Cambodians in Areas Affected by HPAI/H5N1

**DOI:** 10.1371/journal.pone.0006466

**Published:** 2009-07-31

**Authors:** Maria D. Van Kerkhove, Sowath Ly, Javier Guitian, Davun Holl, Sorn San, Punam Mangtani, Azra Ghani, Sirenda Vong

**Affiliations:** 1 Epidemiology Unit, Institut Pasteur in Cambodia, Phnom Penh, Cambodia; 2 Infectious Disease Epidemiology Unit, London School of Hygiene and Tropical Medicine, London, United Kingdom; 3 MRC Centre for Outbreak Analysis & Modelling, Department of Infectious Disease Epidemiology, Imperial College London, London, United Kingdom; 4 Epidemiology Division, The Royal Veterinary College, London, United Kingdom; 5 National Veterinary Research Institute, Department of Animal Health and Production, Ministry of Agriculture, Forestry and Fisheries, Cambodia, Phnom Penh, Cambodia; U.S. Naval Medical Research Center Detachment/Centers for Disease Control, United States of America

## Abstract

**Background:**

Since 2004, 21 highly pathogenic avian influenza H5N1 outbreaks in domestic poultry and eight human cases have been confirmed in Cambodia. As a result, a large number of avian influenza education campaigns have been ongoing in provinces in which H5N1outbreaks have occurred in humans and/or domestic poultry.

**Methodology/Principal Findings:**

Data were collected from 1,252 adults >15 years old living in two southern provinces in Cambodia where H5N1 has been confirmed in domestic poultry and human populations using two cross-sectional surveys conducted in January 2006 and in November/December 2007. Poultry handling behaviors, poultry mortality occurrence and self-reported notification of suspect H5N1 poultry cases to animal health officials in these two surveys were evaluated. Our results demonstrate that although some at risk practices have declined since the first study, risky contact with poultry is still frequent. Improved rates of reporting poultry mortality were observed overall, but reporting to trained village animal health workers decreased by approximately 50%.

**Conclusions/Significance:**

Although some improvements in human behavior have occurred, there are still areas—particularly with respect to the handling of poultry among children and the proper treatment of poultry and the surrounding household environment—that need to be addressed in public health campaigns. Though there were some differences in the sampling methods of the 2006 and 2007 surveys, our results illustrate the potential to induce considerable, potentially very relevant, behavioral changes over a short period of time.

## Introduction

Since 2004, 21 highly pathogenic avian influenza (HPAI) H5N1 outbreaks in domestic poultry have been confirmed in Cambodia including six H5N1 outbreaks in Kampong Cham Province (5 outbreaks) and Prey Veng Province (1 outbreak) [1], [2]. In April 2006 and April 2007, Cambodia's sixth and seventh H5N1 human cases were confirmed in Prey Veng and Kampong Cham provinces, respectively [3], [4]. As a result of the human and domestic poultry H5N1 outbreaks in these two provinces, a large number of avian influenza education campaigns have been carried out in these areas. In January 2006, a cross-sectional survey was conducted in Kampong Cham and Prey Veng Provinces, Southern Cambodia, to determine the extent of backyard poultry ownership and to obtain an in depth understanding of the behavior of adults domestically exposed to poultry [5]. The 2006 study found that despite widespread knowledge on avian influenza, most rural Cambodians undertook high-risk practices when handling poultry [5]. Here we report the results of a second cross-sectional survey conducted in the same two provinces in November-December 2007 to evaluate changes in poultry handling behaviors since the initial survey.

## Methods

Details of the first survey conducted in January 2006 have been published previously [5]. In brief, villages were randomly selected with probability proportional to size (PPS) and households with poultry were selected until 20 individuals per village were interviewed. In the 2007 study, which was carried out in November-December 2007, villages were also chosen using PPS [6]. However we first identified districts in Kampong Cham and Prey Veng with high population density for inclusion based on data from a 1998 census rather than choosing villages from H5N1 related high-risk districts listed by the Food and Agriculture Organization of the United Nations (FAO) as having had training programs for Village Animal Health Workers as was done in the 2006 survey. By the time of the data collection for the 2007 survey, all districts included in this study had village animal health workers trained by the FAO and the National Veterinary Research Institute (NaVRI) of Cambodia.

Details of the data collection used in the 2007 survey are provided elsewhere [6]. Briefly, within each village the first household was chosen randomly from the centre of the village. Subsequent households were then systematically sampled using a sampling interval having been chosen at random (from 1 to 10) for each village until thirty people (i.e., 10 male, 10 female adults [>15 years old] and 10 children [≤15 years old]) plus one village chief were interviewed. Data from children and village chiefs have been presented elsewhere [6]. Two separate standardized closed-ended questionnaires were developed for the heads of household and adult family members. All subjects were asked questions to evaluate contact patterns with domestic poultry and their understanding of avian influenza. In addition, the heads of household questionnaire addressed household poultry and other animal ownership and poultry mortality experienced by the household during the previous 8-month period.

During piloting of the questionnaires in the 2007 survey, we identified substantial difficulties with subjects recalling events over specified periods of time. Subjects found it easier to be reminded of a major event in order to recall events and therefore, we piloted and then asked in the main questionnaire recall periods since a major Cambodian holiday—the Khmer New Year (mid-April annually). Subjects were therefore asked to recall the event or practice within the previous 8-months, i.e., between the time of the interview and the Khmer New Year holiday period (mid-April annually). Mortality was assessed using several questions: 1) *Have you experienced any poultry mortality in your household since the Khmer New Year and if yes,* 2) *how many (chickens or ducks) were sick from illness since the Khmer New Year* and 3) *of those that were sick from illness, how many died since the Khmer New Year.* During the 2006 survey, poultry mortality was evaluated over the previous six-month period.

All responses to poultry contact questions were recorded as binary (yes/no) and frequencies of contact (when evaluated) were recorded as always, sometimes or never. The questionnaire data was checked to assess completeness of questionnaires and errors in data recording by interviewers prior to double entry into EpiData v3.1 (EpiData association, Odense, Denmark).

To evaluate changes in poultry handling behavior and knowledge of avian influenza, we compared the responses of subjects >15 years old living in Kampong Cham (n = 400) and Prey Veng (n = 400) included in the 2007 study with subjects >15 years old from the same two provinces (n = 217, n = 235, respectively) in the 2006 study. The results presented are the differences in responses over the 23-month period between studies.

Demographic differences between subjects >15 years old in 2006 and 2007 were evaluated using chi-square or Fisher's exact tests as appropriate. Differences in poultry mortality experienced, self reported notification of poultry mortality to authorities, self-reported source of avian influenza information and knowledge of avian influenza transmission from poultry to humans were evaluated by province among households that owned poultry using chi squared or Fisher's exact tests, as appropriate. Differences in self-reported poultry handling behaviors between subjects >15 years old in 2006 and 2007 were assessed using chi-square tests or Fisher's exact tests as appropriate. All analyses were adjusted for gender and p-values of <0.05 were considered statistically significant. All statistical analyses were performed using STATA v10 (StataCorp, College Station, Texas).

Ethical approval was granted from the Cambodian Ministry of Health and London School of Hygiene and Tropical Medicine ethical committees. Prior to sampling, field visits were conducted and meetings were held with provincial veterinarians and village chiefs to explain the study objectives and procedures. In the 2007 study, informed written consent was obtained from all subjects or their guardians prior to interview. Verbal consent was obtained from all participants in the 2006 study.

## Results

A total of 452 subjects >15 years old from 23 villages (11 villages in Kampong Cham, 12 villages in Prey Veng) from the 2006 study and 800 subjects >15 years old from 38 villages (19 villages in Kampong Cham and 19 villages in Prey Veng) from the 2007 study were included in the analyses. No villages or persons included in the 2006 survey were included in the 2007 survey. Demographic characteristics of the study subjects from the two surveys are provided in Table 1. Study subjects did not differ by age, education level, house composition or asset ownership. When compared to the 2006 survey results, subjects in the 2007 study were more likely to be male by study design and less likely to classify themselves as “farmers.”

**Table 1:** Demographic characteristics of subjects >15 years old included in the 2006 and 2007 studies, Kampong Cham and Prey Veng, Cambodia.

Characteristics	2006 (n = 452)	2007 (n = 800)	p value†
Gender (% male)	178 (39.4)	401 (50.1)	<0.001
Age (median, IQR)	38 (27–48)	36 (24–49)	
Occupation (% Farmer)	400 (88.5)	557 (70.2)^a^	<0.001
*Education (highest level reached) n (%)*		b	
None	74 (16.4)	170 (21.3)	
Primary	258 (57.1)	413 (51.7)	
Secondary	95 (21.0)	164 (20.5)	
High School	21 (4.7)	40 (5.0)	
Beyond High School	4 (0.9)	4 (0.5)	
Pagoda	NA	8 (1.0)	0.06‡
*Asset Ownership*	d	^e^	
TV	173 (64.6)	133 (66.5)	0.66
Radio	132 (49.2)	96 (48.0)	0.79
Car	1 (0.4)	5 (2.5)	0.09
Bicycle	224 (83.6)	164 (82.0)	0.65
*Poultry Ownership n (%)*	c	d	
Chickens	260 (97.0)	176 (88.0)	<0.001
Ducks	97 (36.2)	82 (41.0)	0.29
Any poultry	89 (33.2)	77 (38.5)	0.24

† *X* ^2^ or Fishers exact test, as appropriate, p-value comparing 2006 vs. 2007.

a n = 794.

b n = 799.

c Assessed only at household level n = 268.

d Assessed only at household level n = 200.

NA Not assessed.

‡ *X* ^2^ test for trend.

Poultry ownership of households included in the 2006 and 2007 study is provided in Table 1. As was found in the 2006 study [5], in the 2007 survey household ownership of poultry (chickens and ducks) was high and flock size was small (median chicken flock size = 17.5 [interquartile range 8–30]; median duck flock size = 7 [interquartile range 4–12]; Table 1). Although, the proportion of households that owned chickens was higher in 2006 vs. 2007 by study design, there were no differences in the proportion of households owning ducks or reporting owning chickens and ducks in the two studies (Table 1).

### Changes in Poultry Mortality and Reporting

Subjects that owned poultry in both Kampong Cham and Prey Veng reported lower poultry mortality in 2007 than in the initial study. There was an 11.2% reduction in reported poultry mortality in households with poultry in Prey Veng (65.5% vs. 54.3%; p = 0.005) and 5.5% reduction in Kampong Cham (48.8% vs. 43.3%; p = 0.19) from 2006 to 2007. Since 2006, reporting of poultry mortality in Kampong Cham and Prey Veng approximately doubled from 7.5% (34/451) in 2006 to 14.4% (55/383) in 2007. Reporting poultry mortality to village chiefs was more than double that noted in the 2006 study (27.8% vs. 61.8%; p = 0.03), however reporting to village animal health workers was lower (23.6% vs. 72.2%, p = 0.002) in 2007 compared to 2006.

**Figure 1:**
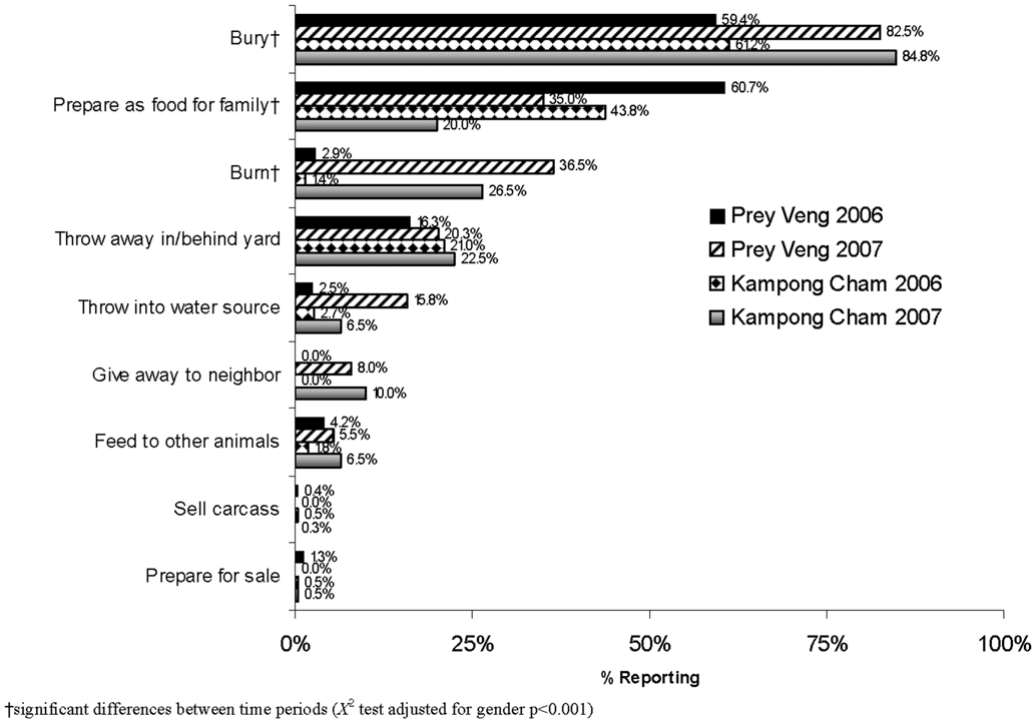
Changes in reported practices of poultry that died from illness in Kampong Cham and Prey Veng Provinces, Cambodia from January 2006 to December 2007. †significant differences between time periods (*X* ^2^ test adjusted for gender p<0.001).

Differences in practices with poultry that died from illness were also noted (Figure 1). There were lower proportions of subjects that reported preparing sick/dead (from illness) poultry for household consumption in both Kampong Cham (p<0.001) and Prey Veng (p<0.001) in 2007 vs. 2006. There were higher proportions of adults that reported burning dead poultry (Kampong Cham p<0.001; Prey Veng p<0.001), burying carcasses (Kampong Cham p<0.001; Prey Veng p<0.001), throwing dead poultry into water sources in Prey Veng (p<0.001) and using dead carcasses to feed other animals in Kampong Cham (p = 0.006) in 2007 when compared to 2006.

### Awareness of Avian Influenza

Awareness of avian influenza was similar in Prey Veng and Kampong Cham in both surveys (>96%), however the self-reported sources of avian influenza information have changed over time (Table 2). When compared to 2006, the proportion of subjects in 2007 reporting that they learned about avian influenza from TV was higher in Prey Veng (+8.3%; p = 0.002) but not so in Kampong Cham (−3.1%; p = 0.06), whereas subjects reported that information about avian influenza from radio was lower in Prey Veng (−7.3%; p<0.001) and higher in Kampong Cham (+6.6%; p = 0.006). There was a higher proportion of subjects reporting their source of avian influenza information from village chiefs (+5.6% in Prey Veng, p = 0.001; +4.5% in Kampong Cham, p = 0.01), and a small apparent decrease from health centers in Prey Veng (−6.2% in Prey Veng; p = 0.002), and village animal health workers (−7.8% in Prey Veng, p = 0.001; −6.4% in Kampong Cham, p = 0.004).

**Table 2:** Changes in source of avian influenza information in Kampong Cham and Prey Veng Provinces, Cambodia from January 2006 to December 2007.

Source of AI Information	Kampong Cham n (%)		p-value†	Prey Veng n (%)		p-value†
	2006	2007		2006	2007	
	n = 210	n = 387		n = 232	n = 394	
Television	166 (79.1)	294 (76.0)	0.06	188 (82.8)	359 (91.1)	0.002
Radio	155 (73.8)	311 (80.4)	0.006	187 (82.4)	296 (75.1)	<0.001
Village chief	2 (0.9)	21 (5.4)	0.01	3 (1.3)	27 (6.9)	0.001
Village veterinary staff	20 (9.5)	12 (3.1)	0.004	27 (11.9)	16 (4.1)	0.001
Health staff/health center	7 (3.3)	18 (4.7)	0.74	21 (9.3)	12 (3.1)	0.002
Newspaper	5 (2.4)	7 (1.8)	0.11	6 (2.6)	4 (1.0)	0.07
Public poster	21 (10.0)	39 (10.1)	0.77	28 (12.3)	51 (12.9)	0.53

† 2006 vs. 2007 by Province *X* ^2^ or Fishers Exact test p-value adjusted for gender.

Knowledge of how avian influenza can be transmitted was higher among adults in Kampong Cham and Prey Veng in 2007 than in January 2006. When compared to the 2006 period, subjects in 2007 more often reported that avian influenza can be transmitted via contact with sick/dead poultry (67.3% vs. 95.6%; p<0.001); slaughtering poultry (45.5% vs. 79.2% p<0.001); and from contact with wild birds (29.7% vs. 86.1%, p<0.001). Fewer subjects in the 2007 study believed that avian influenza can be transmitted via contact with healthy poultry (26.4% in 2006 vs. 11.4% in 2007, p<0.001) in 2007 vs. 2006.

### Changes in Poultry Contact Patterns

Risky behaviors were still frequent in December 2007 (Table 3). However, compared to 2006 lower proportions of subjects reported touching sick/dead poultry with bare hands (p<0.001), using dead domestic poultry from the yard for household consumption (p<0.001); collecting dead wild birds from the field for household consumption (p = 0.002), and using poultry feces for manure (p<0.001). Higher proportions were found of subjects reporting allowing children to play with poultry (p = 0.06) and washing poultry products in water sources (p<0.001).

**Table 3:** Changes in poultry contact in Kampong Cham and Prey Veng provinces, Cambodia from January 2006 to December 2007.

Reported Practice	All Subjects n (%)		
	2006 n = 450	2007 n = 800	p-value‡
*Contact with domestic poultry*			
Touch sick or dead poultry with bare hands	339 (75.3)	337 (42.1)	<0.001
Allow children in the household play (touch and catch) with poultry	92 (20.4)	205 (25.6)	0.06
Use dead domestic poultry from yard for household consumption	203 (45.1)	108 (13.5)	<0.001
Care or help care for poultry	319 (70.6)	588 (73.5)	0.03
Slaughter poultry	173 (38.3)	286 (35.8)	<0.001
*Contact with poultry at live bird markets*			
Ever bought poultry from the market for food during the study period	43 (9.4)	62 (7.8)	0.48
*Contact with wild birds*			
Eat wild birds	149 (33.1)	277 (34.7)	<0.001
Collect dead wild birds from the field for household consumption	37 (8.2)	36 (4.5)	0.002
Ever prepared wild birds for food	114 (31.2)	217 (27.1)	<0.001
*Potential environmental contamination*			
Prepare poultry near a pond, river, or water well	84 (23.0)	220 (27.5)	<0.001
Wash poultry products directly in the water source (pond/river)	6 (1.6)	99 (12.7)	<0.001
Use poultry feces for manure	347 (76.8)	494 (61.8)	<0.001

‡ *X* ^2^ or Fishers exact test p-value adjusted for gender.

## Discussion

We conducted two cross-sectional surveys in Kampong Cham and Prey Veng provinces, Cambodia in January 2006 and November-December 2007 and carried out an ecological comparison of the poultry handling practices among subjects during the two time periods.

Since December 2005, the NaVRI with the assistance of FAO, has funded passive HPAI/H5N1 surveillance systems of domestic poultry in nine of Cambodia's 24 provinces (including Kampong Cham and Prey Veng provinces) using village animal health workers who are trained to identify and report acute high mortality in poultry. Since January 2006, poultry mortality reporting in the study areas has approximately doubled from 7.5% (34/451) to 14.4% (55/383) in 2007. During the 23 months between studies, reporting to the village chief increased by a factor of 2.5 in Kampong Cham and by a factor of 2.1 in Prey Veng. However, reporting to village animal health workers decreased by almost 50% in both provinces. Reasons for this could be because subjects are unaware of whom to report to and therefore report to their village chief, or because poultry are not considered as important as cattle or water buffalo and therefore do not attract the same attention as animals that can bring in more income for the household [2]. This could also be because of a fear that reporting to officials will result in the culling infected animals without providing adequate compensation [2]. Further investigations are therefore needed to evaluate the reason for this decline.

Although awareness of avian influenza was high among all respondents included in this study, understanding of how H5N1 can be transmitted from poultry to humans continues to be low since risky poultry handling behavior remains common in rural areas. Improvements were observed in the reported behavior of adults including the reduction of bare hand contact with sick/dead poultry, collecting dead domestic and wild poultry for food and using poultry feces for manure. Communication strategies in Cambodia have largely focused on improving awareness of “bird flu,” improving basic hygiene practices and reducing risky poultry handling behavior [7]. For example, messages have discouraged touching sick or dead poultry, allowing children to play with poultry or come in contact with areas that may be contaminated with poultry feces or feathers, how to cook poultry safely and clean up food preparation areas and tools, education on how influenza viruses are transmitted, and advice on how to protect themselves against transmission from poultry purchased at markets [7].

Messages have not, however, included methods to reduce poultry contamination in the environment around the home. Recent studies in Cambodia [8], [9] and elsewhere [10] show that environmental exposures could play an important role in the risk of H5N1 infection in children. Practices including washing poultry products directly in the household water source and allowing children play (touch and/or catch) with poultry could result in more human H5N1 cases should the virus recur in domestic poultry.

This analysis is limited by the slightly different study populations as evidenced by demographic differences found between the two study populations. Differences in gender, self-reported occupation and household ownership of chickens were found; however there were no differences in socioeconomic factors (education level and asset ownership), household ownership of any poultry or ducks only. For this reason we consider unlikely that demographic differences between the two study populations can explain the observed differences in levels of poultry handling or poultry mortality reporting practices. Decreases in reporting patterns and the failure of the educational efforts to increase the likelihood of reporting, as shown in our results is critically relevant for disease surveillance since passive HPAI surveillance and therefore the control of infection in rural areas depends on people's willingness to report. Our study suggests that educational efforts that succeed at raising awareness and knowledge about disease transmission and risk for human infection do not succeed in increasing the likelihood of reporting poultry mortality to authorities. Because of this, issues such as compensation have to be carefully considered, especially in Cambodia where compensation for culling is not provided.

Changes in human behavior can facilitate or impede the spread of transmission from one individual or species to the next. Although some improvements in human behavior have been shown, there are still areas—particularly with respect to the handling of poultry among children and the proper treatment of poultry and the surrounding household environment—that need to be addressed in public health campaigns. We believe that these results illustrate the potential to induce considerable, potentially very relevant, behavioral changes over a short period of time.
